# Efficacy of traditional herbal medicine for psychological sequelae in COVID-19 survivors: A protocol for systematic review and meta-analysis

**DOI:** 10.1097/MD.0000000000025609

**Published:** 2021-05-21

**Authors:** Eunjin Kim, Jungyoon Choi, Sang Yeon Min, Ji Hwan Kim, Aram Jeong

**Affiliations:** a Department of Pediatrics of Korean Medicine, Korean Medicine Hospital, Dongguk University Bundang Medical Center; b Department of Pediatrics of Korean Medicine, Graduate School of Dongguk University; c Department of Pediatrics of Korean Medicine, Korean Medicine Hospital, Dongguk University Ilsan Medical Center, Gyeonggi-do; d Department of Sasang Constitutional Medicine, College of Korean Medicine, Gachon University, Seongnam; e Department of Korean Pediatrics, College of Korean Medicine, Gachon University, Gyeonggi-do, Republic of Korea.

**Keywords:** COVID-19, herbal medicine, mental health

## Abstract

**Introduction:**

This study is the protocol for a systematic review to evaluate the efficacy of herbal medicine on COVID-19 survivors with psychological sequelae. Currently, there are many COVID-19 survivors with psychological sequelae as COVID-19 has widely spread all over the world. However, there is no critically appraised evidence of the benefit of herbal medicine for COVID-19 survivors with psychological sequelae.

**Materials and Methods:**

We will search 11 electronic databases from inception to December, 2022: 4 English databases, MEDLINE, PubMed, Excerpta Medica database (EMBASE), and the Cochrane Central Register of Controlled Trials (CENTRAL); 3 Chinese databases, the Chinese National Knowledge Infrastructure, the Chinese Scientific Journal database, and the Wan Fang database; and 4 Korean databases, the Oriental Medical Advanced Searching Integrated System, the Korean Studies Information Service System, the National Digital Science Links, and the Research Information Sharing Service. We will include randomized controlled trials (RCTs), non-RCTs, and quasi-RCTs for all formations of TRADITIONAL herbal medicine versus conventional drug, placebo, and no treatment for COVID-19 survivors. We will only include the COVID-19 survivors with psychiatric symptoms lasting at least 1 month, regardless of their race, sex, and age.

**Discussion and conclusions:**

This systematic review will be published in a peer-reviewed journal. The findings will provide evidence and treatment directions for clinicians. This protocol does not need ethical approval because it will be based on published research.

**Prospero registration number:**

PROSPERO CRD42020210592

## 1 Introduction

Coronavirus disease 2019 (COVID-19), which appeared first in Wuhan in December, 2019 and is caused by severe acute respiratory syndrome coronavirus 2 (SARS-CoV-2), has rapidly spread beyond China to >220 countries and territories.^[1–3]^ The World Health Organization (WHO) declared COVID-19 a “global pandemic” on March 11, 2020.^[4]^ Initially, zoonotic transmission was considered a primary source, and it has since spread via respiratory secretions and increased globalization.^[5]^ As of January 5, 2021, 84,474,195 confirmed cases of COVID-19, including 1,848,704 deaths, have been reported to the World Health Organization, and the pandemic is still ongoing.^[3]^ The US Food and Drug Administration first approved Remdesivir, an inhibitor of the viral RNA polymerases that inhibit SARS-CoV-2 activity in vitro, as a COVID-19 treatment.^[6,7]^ However, this regime has not been universally adopted, and clinical treatment is currently mainly based on patients’ individual symptoms.^[8]^

The COVID-19 pandemic is similar to 2 previous coronavirus infection outbreaks, severe acute respiratory syndrome (SARS) and Middle East respiratory syndrome (MERS), which result in viral pneumonia and acute respiratory distress syndrome.^[5]^ More than one-third of SARS and MERS survivors suffered from neuropsychiatric problems, such as stress, anger, and depression, for >6 months, and impaired quality of life for ≥12 months.^[9,10]^ Based on previous coronavirus infection outbreaks, we must focus on long-term clinical health problems and psychological complications in survivors.^[9]^

Due to physical discomfort, fear of infecting others, loneliness and depression from quarantine, and instability of employment and income, COVID-19 survivors are more likely to suffer a high incidence of psychological distress, including post-traumatic stress disorder (PTSD), anxiety, and depression.^[11]^ Therefore, we must pay attention to psychological sequelae and prepare early intervention strategies, especially for vulnerable survivors with pre-existing illness.^[12]^

Traditional herbal medicine (THM) has been used to treat emotional symptoms and improve quality of life, so it has potential as an adjuvant therapy for COVID-19 survivors’ psychological complications_._^[13–15]^ There have been no systemic reviews focusing on the psychological after-effects of COVID-19 survivors. Therefore, we will evaluate the effects of THM to provide guidelines for clinical practice.

## 2 Materials and methods

Our research was registered at PROSPERO (CRD42020210592) on December 15, 2020. We will also carry out this systematic review according to the Preferred Reporting Items for Systematic Reviews and Meta-Analyses.^[16]^

### 2.1 Data sources

We will search for records deposited up until December 2022 in 11 electronic databases: MEDLINE, PubMed, Excerpta Medica, the Cochrane Central Register of Controlled Trials (CENTRAL) in English, the Chinese National Knowledge Infrastructure, the Chinese Scientific Journal and the Wan Fang Databases in Chinese, the Oriental Medical Advanced Searching Integrated System, the Korean Studies Information Service System, the National Digital Science Links, and the Research Information Sharing Service in Korean. There will be no language restrictions.

Search terms for English databases will be as follows: (COVID-19 OR SARS-CoV-2 OR severe acute respiratory syndrome coronavirus 2 OR 2019-nCoV OR HCoV-19) AND (mental health OR psychological sequela OR psychological health OR anxiety OR depression OR PTSD OR PTSS or post-traumatic stress disorder OR post-traumatic stress symptoms) AND (Chinese medicine OR traditional herbal medicine OR herbal medicine OR Japanese medicine OR traditional medicine OR Oriental medicine OR Korean medicine OR Kampo OR complementary medicine). The search terms will be appropriately translated for use in different databases. A detailed search strategy is shown in Table 1.

**Table 1:** Search Strategy for PubMed in the English databases.

**#1** COVID-19[Title/Abstract]
**#2** SARS-CoV-2 [Title/Abstract]
**#3** 2019-nCoV[Title/Abstract]
**#3** Severe acute respiratory syndrome coronavirus 2 [Title/Abstract]
**#4** HCoV-19 [Title/Abstract]
#5 #1 OR #2 OR #3 OR #4
#6 Chinese medicine [MeSH Terms]
#7 traditional herbal medicine [MeSH Terms]
#8 herbal medicine [MeSH Terms]
#9 Japanese medicine[Title/Abstract]
#10 Traditional medicine [Title/Abstract]
#11 Oriental medicine [MeSH Terms]
#12 Kampo [MeSH Terms]
#13 Korean medicine [MeSH Terms]
#14 Complementary medicine [MeSH Terms]
#15 #6 OR#7 OR #8 OR #9 OR #10 OR #11 OR #12 OR #13 OR #14
#16 mental health [Title/Abstract]
#17 psychological sequela [Title/Abstract]
#18 psychological health [Title/Abstract]
#19 anxiety [Title/Abstract]
#20 depression [Title/Abstract]
#21 PTSD [Title/Abstract]
#22 PTSS [Title/Abstract]
#23 post-traumatic stress∗ [Title/Abstract]
#24 #16 OR #17 OR #18 OR #19 OR #20 OR #21 OR #22 OR #23
#25 #5 AND #15 AND #24

### 2.2 Study selection

#### 2.2.1 Types of studies

We will include all types of clinical studies (eg, randomized controlled trial [RCT], non-RCT, and quasi-RCT) that assessed the efficacy of THM, regardless of blinding, language, and types of reporting. We will exclude kinds of case reports and studies that do not include full text availability.

#### 2.2.2 Types of participants

We will only include COVID-19 survivors, not COVID-19 patients. Only COVID-19 survivors with psychiatric sequelae who have been diagnosed for at least 1 month will be included. The participants should have psychological disorders, such as those diagnosed by international diagnostic criteria (eg, the American Psychiatric Association's DSM-5, and the *ICD-10*) regardless of age, sex, and race.

#### 2.2.3 Types of interventions

We will include clinical studies that used all types of THM as experimental interventions. All herbal formulations will be allowed. There will be no limitations on the type of formulation (eg, decoction, tablets, capsules, pills, powders, and extracts), dosage, duration of intervention, number of herbs, and administration methods. The comparators are patients who have been treated with interventions such as conventional drug treatments, placebos, and no treatment.

### 2.3 Outcome measures

One of the following outcome measures will be used. The primary outcomes will be the therapeutic effects of herbal medicine as measured by one of the following anxiety, depression, insomnia, distress, and fear-related questionnaires: psychological stress questionnaire, anxiety scale, depression score, dream anxiety score, and short form 36 health survey questionnaire. The total effective rate will also be included. This is the number of patients who showed an improvement in psychological symptoms among all patients. These outcome measures are generally expressed as follows: “cured,” “markedly improved,” “improved,” “slightly better”, and “no effect”. The secondary outcomes will be evaluated based on adverse events.

### 2.4 Data collection and analysis

#### 2.4.1 Selection of studies

**Figure 1:**
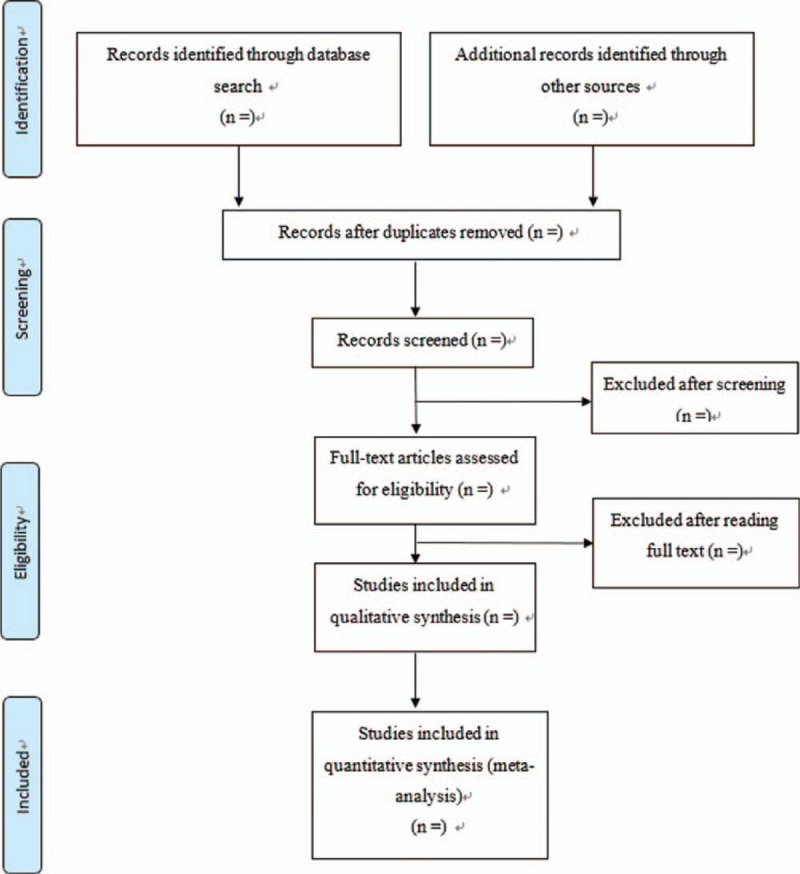
PRISMA flow chart outlining the search process.

Two independent authors (EJK and ARJ) will extract data from the included articles according to predefined criteria. A flowchart outlining the search strategy is shown in Figure 1. Disagreements will be resolved through discussions between all of the authors.

#### 2.4.2 Data extraction and quality assessment

All articles will be read fully by two investigators adhering to predesigned forms that include study design, participant's general information, outcome measures, and results. Any disagreement regarding data extraction will be resolved by discussion with a third reviewer. Risk of bias will be assessed using the Cochrane Handbook risk of bias assessment tool version (V.5.1.0). These criteria include the following categories: allocation concealment, random sequence generation, blinding of participants and personnel, blinding of outcome measures, incomplete outcome data, selective reporting, and other risk of biases.^[17]^ Each study will be assigned as “unclear risk,” “low risk”, or “high risk” of bias. For articles in which the information is insufficient, we will try to contact the authors via phone or email to obtain complete information.

#### 2.4.3 Data synthesis

For continuous data, mean differences (MDs) with 95% confidence intervals (CIs) will be used to measure the effects of treatment. If the outcome measures use different scales, we will use standard MDs with 95% CIs. For dichotomous data, we will abstract relative risks (RRs) with 95% CI. We will calculate the weighted MDs, relative risks, and standard MDs with 95% CIs using Review Manager Version 5.4 software for Windows (Cochrane, London, UK).

#### 2.4.4 Addressing missing data

For articles in which sufficient information is not provided, we will try to contact the authors via phone or email to obtain complete information. We will include the results of the intention-to-treat analysis. For data on missing data in clinical trial results, last observation carried-forward analysis will be performed. The impact of missing data will be described in this review.

#### 2.4.5 Assessment of heterogeneity

Heterogeneity among trials will be identified by the I^2^ and Chi-squared test statistics. If the included studies have high heterogeneity (*I*^2^ > 50%), we will use a random-effects model for pooling data across studies. Otherwise, a fixed-effects model will be used.

#### 2.4.6 Assessment of publication bias

To evaluate publication bias, we will construct a funnel plot using Cochrane software if the number of included studies is sufficient (>10 studies). A symmetrical funnel plot indicates no possibility of publication bias, whereas an asymmetrical funnel plot indicates a high possibility of publication bias. If we identify publication bias through analysis of the funnel plot, we will discuss possible reasons such as small-study effects.

#### 2.4.7 Sensitivity analysis

To confirm the robustness of the review finding, a sensitivity analysis will be conducted after excluding poor quality studies, outliers, and missing values. We will compare original with sensitivity analysis results.^[17]^

#### 2.4.8 Subgroup analysis

If there are sufficient data or significant heterogeneity, we will conduct subgroup analyses according to the following: age, treatment period, type of control, duration of disease, type of psychological sequela, and symptoms.

#### 2.4.9 Assessment of evidence quality

We will use the Grading of Recommendations Assessment, Development, and Evaluation approach to conduct a quality assessment for each outcome.^[18]^ The quality of evidence will be recorded as “high,” “moderate,” “low,” or “very low.”

#### 2.4.10 Ethics

Ethical approval is not required since this review is based on published research.

## 3 Discussion

The world has undergone 3 threats to global health resulting from coronaviruses: SARS, MERS, and COVID-19. COVID-19 infection is still increasing rapidly, despite countermeasures in individual countries, such as China's lockdown strategy, Sweden's herd immunity strategy, Taiwan's immigration and facial strategy, and Korea's social distancing and facial mask strategy.^[19]^

According to previous studies, respiratory viral outbreaks can cause various types of neuropsychiatric symptoms such as affective disorder, sleep disorder, and suicidality.^[20–22]^ COVID-19 can also cause delirium, depression, anxiety, fatigue, and PTSD^[23]^; therefore, suitable treatment to manage psychological sequelae during the recovery period must be available. THM has been used for psychological problems after acute respiratory disease, such as SARS,^[24]^ and natural disasters or significant events,^[25,26]^ such as PTSD of survivors after the Sichuan earthquake^[27]^ and the Great East Japan Earthquake and Tsunami.^[28]^ THM affects immune effector cells such as macrophages, monocytes, B cells, neutrophils, and mast cells to modulate the immune system and maintain homeostasis.^[29–32]^ It also controls neuroreceptor binding and channel transporter activity by modulating neuronal communication or the hypothalamic-pituitary-adrenal axis. Therefore, some herbal medicines can be used as antidepressants by sensitizing serotonin receptors or inhibiting monoamine oxidase.^[33,34]^

The aim of this systemic review is to evaluate the effects of THM on psychological sequelae in COVID-19 survivors. However, it has some limitations. First, it is difficult to conduct clinical trials, so we will also include non-RCTs, which might induce low reliability in the conclusions. Second, the pandemic is ongoing and the databases are continually being updated. To the best of our knowledge, this will be the first systemic review focusing on COVID-19 survivors’ psychological sequelae, and it could provide evidence and treatment directions for clinicians.

## Author contributions

EJK and ARJ conceptualized and designed the study. ARJ and JHK developed the search strategy. EJK, ARJ and JYC drafted the manuscript. SYM interpreted and revised this article. All authors read and approved the final manuscript.

**Conceptualization:** Eunjin Kim, Aram Jeong.

**Funding acquisition:** Aram Jeong.

**Methodology:** Ji Hwan Kim.

**Supervision:** Aram Jeong.

**Validation:** Sang Yeon Min.

**Writing – original draft:** Eunjin Kim, Jungyoon Choi, Aram Jeong.
